# Machine Learning-Based Detection of *Anaplasma* spp. Using Dielectric Properties of Host Cells

**DOI:** 10.3390/pathogens15070765

**Published:** 2026-07-21

**Authors:** Hossein Valishirin, Sai Deepika Reddy Yaram, Negar Farhang Doost, Soumya K. Srivastava, Shira L. Broschat

**Affiliations:** 1 School of Electrical Engineering and Computer Science, Washington State University, Pullman, WA 99164, USA; hossein.valishirin@wsu.edu; 2 Department of Chemical and Biomedical Engineering, West Virginia University, Morgantown, WV 26506, USA; sy00021@mix.wvu.edu (S.D.R.Y.); nf00011@mix.wvu.edu (N.F.D.); 3 Department of Microbiology, Immunology, and Cell Biology, West Virginia University, Morgantown, WV 26506, USA; 4 Department of Veterinary Microbiology and Pathology, Washington State University, Pullman, WA 99164, USA; 5 Paul G. Allen School for Global Health, Washington State University, Pullman, WA 99164, USA

**Keywords:** *Anaplasma phagocytophilum*, anaplasmosis, dielectric signatures, label-free diagnostics, machine learning, tick-borne infections

## Abstract

Tick-borne bacterial infections such as those caused by *Anaplasma phagocytophilum* are difficult to diagnose, particularly at early stages, because they rely on specialized laboratory tests. Dielectric-based measurements provide a label-free way to probe cellular state and may offer useful information. In this study, we explored whether *A. phagocytophilum*-infected and uninfected HL-60 human promyelocytic leukemia cells can be distinguished using dielectric measurements collected under controlled in vitro conditions. Measurements were performed at two medium conductivities (100 and 300 µS/cm), and three dielectric properties were examined: cytoplasmic conductivity, specific membrane conductance, and specific membrane capacitance. We used exploratory analysis, statistical tests, and interpretable linear classifiers, with performance evaluated using leave-one-out cross-validation. Membrane-related properties, especially specific membrane conductance, showed the clearest separation between infection states and were consistently the most informative across models. Both classification performance and effect sizes were stronger at the higher medium conductivity condition. These results are encouraging, but because the sample size was small and consisted only of technical replicates, they should be interpreted as preliminary measurement-level evidence. This study is intended as a pilot and supports further work with larger datasets, additional cell types, and broader experimental conditions.

## 1. Introduction

Tick-borne bacterial infections have emerged as a growing public health concern worldwide, with their incidence and geographic distribution expanding steadily over the past several decades [1,2]. Among these, human granulocytic anaplasmosis (HGA), caused by the obligate intracellular bacterium *Anaplasma phagocytophilum*, is recognized as a clinically significant and potentially life-threatening disease [3,4]. *A. phagocytophilum* is transmitted primarily through the bite of *Ixodes* spp. ticks and preferentially infects neutrophils, where it replicates within a membrane-bound vacuole in the host cell cytoplasm [5,6]. The clinical presentation of HGA is often nonspecific, characterized by fever, headache, myalgia, and malaise, which overlap considerably with other febrile illnesses [3,7]. Current laboratory diagnosis methods include polymerase chain reaction (PCR), indirect immunofluorescence assay (IFA), and microscopic identification of morulae in peripheral blood smears [8,9]. However, PCR sensitivity can be limited prior to bacteremia, serological tests based on antibody detection frequently yield negative results during the first 7–10 days of illness, and blood smear examination lacks adequate sensitivity for routine clinical use [8,10]. Furthermore, confirmatory serological diagnosis requires the collection of paired acute and convalescent serum samples, introducing delays that are incompatible with timely clinical decision-making [9]. These diagnostic limitations highlight the need for alternative, rapid, and label-free approaches that can detect infection-associated changes at the cellular level without relying on molecular amplification, antibody responses, or complex sample preparation.

The dielectric properties of biological cells, including membrane capacitance, membrane conductance, and cytoplasmic conductivity, are intrinsically linked to cellular morphology, membrane composition, and intracellular physiology [11,12]. When subjected to alternating electric fields, the polarization response of a cell is governed by the insulating nature of the phospholipid bilayer and the conductive characteristics of the cytoplasm, which together give rise to frequency-dependent spectra that can be modeled using single-shell dielectric frameworks [13,14]. These electrical parameters have been shown to serve as sensitive indicators of cellular state, with measurable changes reported during processes such as apoptosis, differentiation, and drug-induced membrane perturbation [15,16,17]. Importantly, intracellular bacterial infection is expected to alter host cell dielectric properties through multiple mechanisms. *A. phagocytophilum* resides within a host cell-derived parasitophorous vacuole that it extensively remodels by redirecting membrane traffic from recycling endosomes, the trans-Golgi network, and multivesicular bodies [5,18,19]. This process involves the recruitment of specific Rab GTPases, a family of small monomeric GTP-binding proteins that act as master regulators of intracellular membrane trafficking in eukaryotic cells [20], and intermediate filament proteins to the vacuolar membrane, as well as alterations in host cell cholesterol uptake and cytoskeletal organization [6,21]. Such modifications to membrane architecture, intracellular compartmentalization, and ion homeostasis are anticipated to produce detectable shifts in cellular dielectric signatures. Indeed, prior studies in the context of malaria, a parasitic disease caused by mosquitoes, have demonstrated that parasitized erythrocytes exhibit significantly altered membrane conductance and capacitance compared with uninfected cells, as measured by electrorotation and dielectrophoretic techniques [22,23]. Building on our prior work, we have established dielectrophoretic crossover frequency analysis as a versatile platform for cellular characterization across multiple domains: infectious disease (distinguishing *Rickettsia*-infected from healthy Vero cells, characterizing *Babesia bovis*), environmental stress responses (microgravity-induced dielectric alterations in erythrocytes and yeast cells), metabolic effects (glucose-mediated changes in *Candidozyma auris* polarizability), clinical diagnostics (temperature and storage duration impacts on erythrocyte properties), and cancer biology (dielectric signatures of late-stage breast carcinoma immune cells) [24,25,26,27,28,29]. However, the application of dielectric profiling to detect intracellular bacterial infections in nucleated host cells has remained largely unexplored. The present study was therefore designed to investigate whether dielectric measurements can distinguish between *A. phagocytophilum*-infected and uninfected HL-60 human promyelocytic leukemia cells under controlled in vitro conditions, employing exploratory analysis, statistical testing, and interpretable machine learning classifiers to assess the discriminatory potential of these dielectric features.

Previous studies have shown that dielectric-based measurements are sensitive to changes in cellular state and membrane properties [30,31]. Most of this work, however, has focused on population-level analyses, single experimental settings, or descriptive results without directly assessing classification performance. As a result, it is still unclear how well dielectric features can distinguish infected cells across different medium conductivities, especially when using interpretable models and limited sample sizes.

In this study, we investigate whether the dielectric properties of HL-60 cells can be used to distinguish *A. phagocytophilum*-infected cells from uninfected controls under two controlled medium conductivity conditions. Measurements of cytoplasmic conductivity, specific membrane conductance, and specific membrane capacitance were analyzed using exploratory visualization, statistical testing, and simple linear classifiers. Performance was evaluated using leave-one-out cross-validation to assess feasibility in a data-limited setting.

The objective of this study was to evaluate, under controlled in vitro conditions, whether dielectric measurements can provide preliminary measurement-level separation between *A. phagocytophilum*-infected and uninfected HL-60 cells. Because the dataset is limited to technical replicates from a small number of biological preparations, the analysis is framed as an exploratory feasibility assessment rather than a definitive validation study.

## 2. Materials and Methods

### 2.1. Cell Culture and Infection Protocol

The HL-60 human promyelocytic leukemia cell line, originally derived from a female patient with acute myeloid leukemia [32], was used in this study. HL-60 cells were selected because they are an established in vitro model for studying *A. phagocytophilum* infection in human granulocytic cells. Primary human neutrophils would be biologically closer to the natural host cell, but their short lifespan and limited capacity for sustained culture make them less suitable for the multi-day infection and measurement workflow used here. Because HL-60 cells are leukemia-derived, their baseline dielectric properties may differ from those of primary neutrophils; therefore, future studies using primary human granulocytes, where feasible, will be needed to confirm the generalizability of these findings. Frozen stocks of both uninfected and *A. phagocytophilum*-infected HL-60 cells were obtained from Dr. Kelly Brayton’s laboratory at Washington State University and stored in liquid nitrogen (−196 °C) until use. Both infected and uninfected cell populations underwent the same cryopreservation and thawing workflow, so any potential freeze–thaw effects would be expected to affect both groups similarly. Therefore, the comparative analysis between infected and uninfected cells was performed under matched cell-preparation conditions. Cells were maintained in RPMI-1640 medium supplemented with L-glutamine (Thermo Fisher Scientific, Waltham, MA, USA) and 10% heat-inactivated fetal bovine serum (FBS) (Thermo Fisher Scientific, Waltham, MA, USA). Complete medium was stored at 4 °C and prewarmed to 37 °C before use [33].

For culturing, the frozen HL-60 cells were rapidly thawed in a 37 °C water bath and transferred to a centrifuge tube. Cells were pelleted at 210× *g* for 10 min at room temperature, and the supernatant was carefully discarded. The resulting pellet was resuspended in 5 mL of fresh complete RPMI medium and transferred to a 25 cm^2^ vented tissue culture flask (VWR, Radnor, PA, USA). Cultures were maintained in a humidified incubator at 37 °C with 5% CO_2_.

Cell density and viability were assessed during routine maintenance using trypan blue (Thermo Fisher Scientific, Waltham, MA, USA) exclusion and a hemocytometer (C-Chip DHC-N01, INCYTO, Cheonan, Republic of Korea). Briefly, 10 µL of well-mixed HL-60 cell suspension was combined with 10 µL of trypan blue, and the mixture was loaded into the hemocytometer. Live cells were counted in four large squares under a microscope, and the total viable cell concentration was calculated using Equation (1).

(1) $$\frac{\left(total\;live\;cells\;counted\times dilution\;factor\times 10,000\right)}{\left(number\;of\;large\;squares\;counted\right)}$$

Cultures were passaged when the cell density reached approximately 5 × 10^5^ to 1 × 10^6^ cells/mL. The volume of cell suspension required to seed a new culture at 1 × 10^5^ cells/mL was calculated using Equation (2).

(2) $$\frac{\left(1\times 10^{5}\right)}{\left(\frac{number\;of\;cells}{mL}from\;hemocytometer\right)}$$

For passage, the cell suspension was transferred to a 15 mL conical tube, diluted with the appropriate volume of fresh prewarmed medium, and returned to a new culture flask for continued incubation [34].

To establish infected cultures, uninfected HL-60 cells were first adjusted to a density between 5 × 10^5^ and 1 × 10^6^ cells/mL. A vial of frozen *A. phagocytophilum*-infected cells was thawed in a 37 °C water bath for 60 s and centrifuged at 2300× *g* for 10 min at room temperature. After removal of the supernatant, the pellet was resuspended in 5 mL of uninfected HL-60 cells at a concentration of 1 × 10^5^ cells/mL. The suspension was mixed gently and transferred to a 25 cm^2^ vented tissue culture flask, followed by incubation at 37 °C in 5% CO_2_.

Infection progression was monitored every two days by cytospin preparation and staining. For infection assessment, microscope slides were assembled in a Shandon Cytospin II Cytocentrifuge (Thermo Fisher Scientific, Waltham, MA, USA) with filter cards and cytofunnels (VWR, Radnor, PA, USA). The infected HL-60 suspension was gently mixed, and 150 µL of cell suspension was loaded into each cytofunnel. Samples were centrifuged at 1000× *g* for 5 min, and the slides were then removed, air-dried, and stained using a Diff-Quik staining kit (Electron Microscopy Sciences, Hatfield, PA, USA). After staining, the slides were examined under the microscope, and 100 cells were counted to determine the percentage of infected cells.

When heavy infection was observed, infected cultures were passaged by transferring a small aliquot of infected suspension (~100–200 µL) into fresh prewarmed RPMI medium containing uninfected HL-60 cells. The cultures were gently mixed and returned to the incubator at 37 °C with 5% CO_2_. Infection levels were monitored continuously, and passaging was repeated as needed to maintain the infected culture.

### 2.2. Dielectric Measurement Setup

Dielectrophoresis (DEP), first described by H. A. Pohl in 1951, is the motion of polarizable particles in a non-uniform electric field [35]. Unlike electrophoresis, DEP is independent of the net charge of the particle and instead depends on differences in dielectric signatures between the particle and the surrounding medium [36,37,38]. Because of this property, DEP is widely used for particle and cell manipulation in microfluidic platforms [39,40,41]. For a spherical particle, the DEP force is given by Equation (3).

(3) $$F_{DEP}=2\pi \varepsilon_{0}\varepsilon_{m}r^{3}Re[K(w)]\nabla E^{2}$$

where (*r*) is the particle radius, $\varepsilon_{m}$ and $\varepsilon_{0}$ are the permittivities of the medium and vacuum, respectively, Re[K(w)] is the real part of the Clausius–Mossotti (CM) factor, and $\nabla E^{2}$ is the electric field gradient. The sign of the CM factor determines the DEP response: positive DEP (pDEP) drives particles toward regions of high electric field, whereas negative DEP (nDEP) moves them away [42]. The CM factor is defined as Equation (4).

(4) $$Re\left[K\left(w\right)\right]=\frac{\left(\varepsilon_{p}^{*}-\varepsilon_{m}^{*}\right)}{\left(\varepsilon_{p}^{*}+{2\varepsilon }_{m}^{*}\right)}$$

where $\varepsilon_{p}^{*}$ and $\varepsilon_{m}^{*}$ are the complex permittivities of the particle and medium, respectively.

To describe cellular dielectric behavior, the single-shell model was used in this study [43]. This model represents the cell as a conductive cytoplasmic core surrounded by a thin membrane and provides a simplified and effective framework for analyzing DEP responses [13,44]. By fitting experimental data to the single-shell model, the dielectric spectrum of the cell can be obtained as a function of frequency and CM factor. In this spectrum, the crossover frequency corresponds to the point at which the DEP force is zero, marking the transition from nDEP to pDEP or *vice versa*. The first crossover frequency is primarily influenced by cell morphology, geometry, and cell membrane properties, as well as by the conductivity of the surrounding medium, whereas the second crossover frequency is more strongly associated with intracellular properties and is less dependent on the medium conductivity [28]. These features make DEP advantageous over purely size-based separation methods, as it enables discrimination based on both cellular morphology and electrical properties [29,39].

For dielectric characterization, a low-conductivity suspending medium was prepared to minimize Joule heating while maintaining suitable osmotic balance, low viscosity, and low cytotoxicity [45]. Briefly, 8.5 g sucrose and 0.3 g dextrose were dissolved in 100 mL of deionized water. Conductivity was adjusted to approximately 100 or 300 µS/cm using 1 × PBS and verified with a conductivity meter (InLab 731-ISM, Mettler Toledo, Columbus, OH, USA). Uninfected and infected HL-60 cell pellets were washed and resuspended in this medium, and all experiments were performed at room temperature (21 °C).

DEP spectral measurements were performed using a 3DEP dielectrophoretic cytometer with a DEPwell 806 chip (DepTech, Uckfield, UK). Prior to sample loading, the chip was rinsed with DEP medium, and the cell suspension was introduced using a 1 mL syringe fitted with a 25G needle (Air-Tite Products Co., Inc., Virginia Beach, VA, USA). A glass coverslip was placed over the chip to reduce meniscus formation before insertion into the analyzer. Measurements were acquired at 10 Vpp over 0.5 kHz to 45 MHz, with a 60 s run time and 30 s analysis period. The medium conductivity was maintained at 100 or 300 µS/cm, and cell radii were obtained from flow cytometry measurements to support accurate dielectric analysis of infected and uninfected cells.

### 2.3. Dataset Description

The dataset used in this study comprises measurements obtained from HL-60 human promyelocytic leukemia cells under controlled in vitro conditions. The dataset was collected to support the feasibility analysis of classifying *A. phagocytophilum* infection based on the electrical properties of individual cells, measured using established dielectric measurement techniques.

A total of 20 dielectric measurements were collected, evenly distributed across the two experimental medium conductivity conditions (100 and 300 µS/cm). It is important to note the replicate structure of this dataset. For each conductivity condition, five technical replicates of *A. phagocytophilum*-infected and five of uninfected HL-60 cell suspensions were analyzed, yielding a balanced dataset with respect to infection status and medium conductivity. These technical replicates, therefore, characterize the reproducibility of the measurement process rather than independent biological variability, as no additional biological replicates were included. Consequently, all subsequent analyses should be interpreted as quantifying measurement-level separability between infected and uninfected samples under the tested conditions, and not as estimates of biologically generalizable differences. We retain this dataset for a proof-of-concept feasibility assessment, while recognizing that a minimum of three independent biological replicates per condition is required to establish biological significance. Population-level dielectric properties were extracted from each replicate by applying a single-shell dielectric model with least-squares curve fitting, as implemented in the 3DEP system.

The following features were extracted for each replicate:

- Cyto Conductivity (S/m): Cytoplasmic conductivity, reflecting the ionic content of the cell interior.
- Sp Mem Conductance (S/m^2^): Specific membrane conductance, indicative of the ionic permeability of the membrane.
- Sp Mem Capacitance (F/m^2^): Specific membrane capacitance, associated with the ability of the cell membrane to store charges.

A categorical variable (Conductivity) specifies the medium conductivity condition for each measurement (100 or 300 µS/cm). The target variable (Infection) is binary, with 0 representing uninfected cells and 1 indicating cells infected with *A. phagocytophilum*.

No missing values were present in the dataset, and no normalization or transformation procedures were applied prior to analysis. All measurements were used in their original form to preserve the natural variability inherent in recordings.

### 2.4. Exploratory and Machine Learning Analysis

An exploratory analysis was performed to examine the distributions and relationships of the dielectric features prior to modeling. Because the dataset is small and consists of dielectric measurements, the analysis focuses on visualization rather than formal inference. For each conductivity condition (100 and 300 µS/cm), boxplots and violin plots were used to compare feature distributions between infected and uninfected cells, while scatter plots and correlation heatmaps were used to examine relationships among features. These visualizations were used to identify general patterns and to guide the subsequent analysis.

Logistic regression and a linear support vector machine (SVM) were used to examine whether dielectric features contained information relevant to distinguishing infected from uninfected cells. The task was formulated as a binary classification problem at the population-level dielectric measurements using cytoplasmic conductivity, specific membrane conductance, and specific membrane capacitance as input features. Models were trained separately for each conductivity condition.

Because the dataset is small, model performance was evaluated using leave-one-out cross-validation (LOOCV). Results are interpreted descriptively to illustrate trends rather than definitive predictive performance.

## 3. Results and Discussion

**Figure 1:**
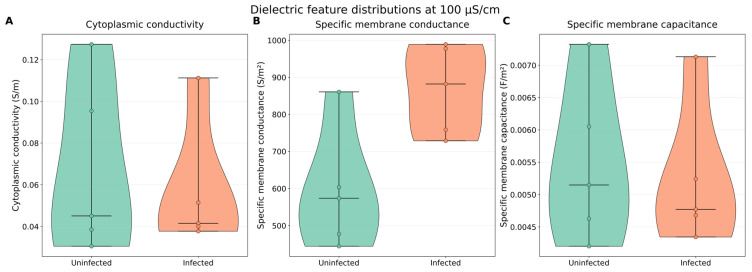
Distribution of dielectric features for uninfected and *A. phagocytophilum*-infected HL-60 samples at 100 µS/cm medium conductivity. Violin plots compare (**A**) cytoplasmic conductivity (S/m), (**B**) specific membrane conductance (S/m^2^), and (**C**) specific membrane capacitance (F/m^2^) between infection groups. The embedded box in each violin plot represents the interquartile range and median, and the width of each violin indicates the relative distribution of the technical replicate measurements.

**Figure 2:**
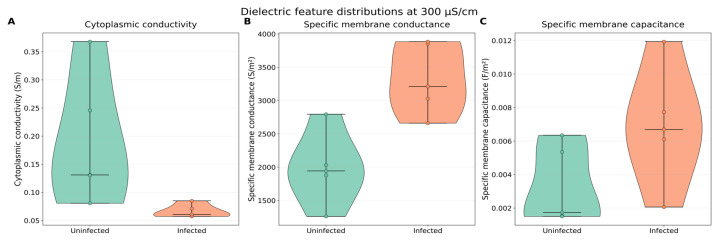
Distribution of dielectric features for uninfected and *A. phagocytophilum*-infected HL-60 samples at 300 µS/cm medium conductivity. Violin plots compare (**A**) cytoplasmic conductivity (S/m), (**B**) specific membrane conductance (S/m^2^), and (**C**) specific membrane capacitance (F/m^2^) between infection groups. The embedded box in each violin plot represents the interquartile range and median, and the width of each violin indicates the relative distribution of the technical replicate measurements.

Figure 1 and Figure 2 show the distributions of cytoplasmic conductivity, specific membrane conductance, and specific membrane capacitance for infected and uninfected samples at 100 µS/cm and 300 µS/cm, respectively. Within each violin plot, the embedded box indicates the interquartile range, the central line represents the median, and the whiskers show the range of observed values. At 100 µS/cm, infected samples generally show lower and less variable cytoplasmic conductivity than uninfected samples, while specific membrane conductance shows the clearest separation, with the infected samples shifted toward higher values. Specific membrane capacitance shows a modest decrease in infected samples, although a substantial overlap remains between groups. Overall, at this conductivity condition, infection is associated most strongly with increased membrane conductance, with smaller changes in cytoplasmic conductivity and membrane capacitance.

At 300 µS/cm, the separation between infected and uninfected samples is more apparent across all three dielectric features. Cytoplasmic conductivity is substantially lower and more tightly clustered in infected samples, while uninfected samples show a broader distribution with higher values. Specific membrane conductance also shows a clear upward shift in infected samples, with limited overlap between groups. Specific membrane capacitance is higher in infected samples as well, although the separation is less distinct than for cytoplasmic conductivity and membrane conductance. Overall, at this medium conductivity, cytoplasmic conductivity and specific membrane conductance appear to be the strongest discriminators of infection status.

To provide exploratory statistical support for the visual patterns in Figure 1 and Figure 2, we performed two-sided Mann–Whitney U tests for each dielectric feature at each conductivity condition. In each comparison, the U statistic summarizes the rank-based separation between the two groups, five infected and five uninfected technical replicates. U ranges from 0 to 25, with values near the middle indicating greater overlap and values closer to either extremes indicating stronger group separation. Effect sizes were summarized using Cohen’s d and rank-biserial correlation, with bootstrap 95% confidence intervals. Because the dataset consists of technical replicates from a single biological preparation per condition, these statistics are interpreted as measurement-level separation rather than evidence of biological generalizability. The results are summarized in Table 1. At 100 µS/cm, specific membrane conductance showed the strongest exploratory separation between infected and uninfected samples, with higher values in infected samples, although this comparison did not remain significant after Benjamini–Hochberg adjustment. At 300 µS/cm, stronger separation was observed, with lower cytoplasmic conductivity and higher specific membrane conductance in infected samples; both comparisons remained significant after adjustment. Specific membrane capacitance also showed a higher value in infected samples at 300 µS/cm, but its adjusted *p*-value was slightly above 0.05.

**Figure 3:**
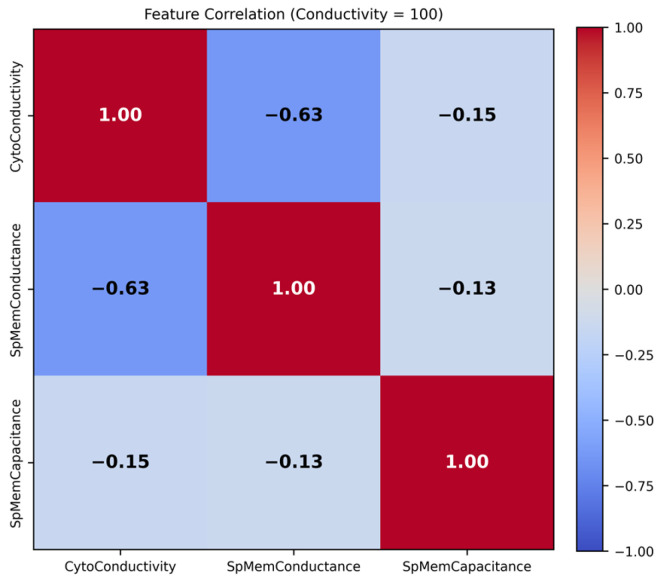
Correlation matrix of dielectric features measured at 100 µS/cm medium conductivity. Values represent pairwise Pearson correlation coefficients among cytoplasmic conductivity, specific membrane conductance, and specific membrane capacitance across the technical replicate measurements. Positive values indicate direct associations, whereas negative values indicate inverse associations between features.

**Figure 4:**
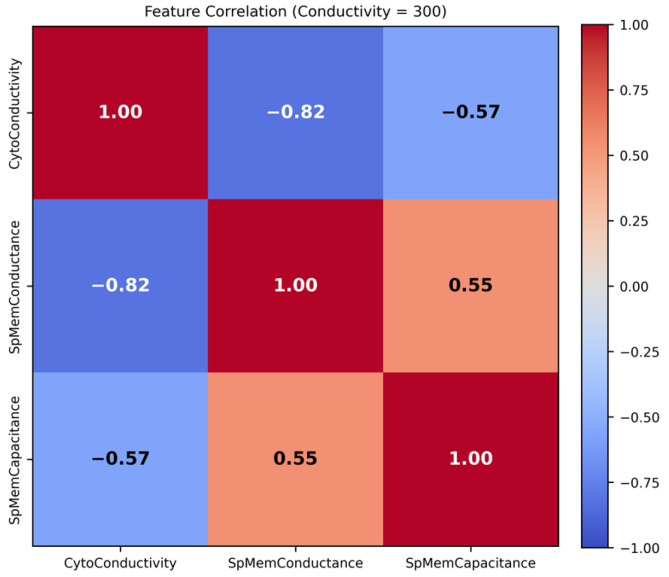
Correlation matrix of dielectric features measured at 300 µS/cm medium conductivity. Values represent pairwise Pearson correlation coefficients among cytoplasmic conductivity, specific membrane conductance, and specific membrane capacitance across the technical replicate measurements. Positive values indicate direct associations, whereas negative values indicate inverse associations between features.

Correlation heatmaps for each conductivity condition are shown in Figure 3 and Figure 4. At 100 µS/cm, cytoplasmic conductivity and membrane conductance show a moderate negative correlation (−0.63), while correlations involving membrane capacitance remain weak. At 300 µS/cm, these relationships become stronger, with a pronounced negative association between cytoplasmic conductivity and membrane conductance (−0.82) and a moderate positive correlation between membrane conductance and membrane capacitance (0.55).

Together, the two correlation matrices suggest a biologically plausible pattern in how cytoplasm and membrane electrical properties may be coordinated as overall conductivity increases. At lower conductivity (100 µS/cm), cytoplasm conductivity and specific membrane conductance are moderately inversely related, while membrane capacitance remains largely independent, implying that early or milder physiological changes primarily affect ion transport between the cytoplasm and membrane without substantially altering membrane structure. In contrast, at higher conductivity (300 µS/cm), the relationships strengthen and broaden; cytoplasm conductivity becomes strongly and negatively coupled to membrane conductance, and membrane capacitance becomes significantly correlated with both, indicating that cytoplasmic ionic reorganization, membrane permeability, and membrane structural properties may be changing together. Biologically, this pattern is consistent with a progression from localized functional changes, such as altered ion channel or transport activity, to more global cellular remodeling, where membrane integrity, surface properties, and intracellular ionic balance become more tightly linked.

The observed coupling between dielectric parameters is consistent with coordinated alterations in host cell physiology induced by *A. phagocytophilum* infection. Cytoplasmic conductivity is governed primarily by intracellular ion concentrations and is regulated by plasma membrane ion channels and transporters [46,47]. Membrane conductance reflects the passive ionic permeability of the plasma membrane, determined by ion channel activity and membrane integrity [48,49]. A negative correlation between these parameters is therefore biologically plausible; increased membrane permeability can promote ionic efflux reducing intracellular ion content and lowering cytoplasmic conductivity. Similar inverse relationships have been reported during apoptosis, where increased membrane leakage coincides with cytoplasmic ion loss [43,50,51]. Given that *A. phagocytophilum* infection has been associated with membrane perturbation and pro-apoptotic changes in HL-60 cells, the strengthening of this inverse relationship at 300 µS/cm is consistent with infection-related changes in membrane transport and intracellular ion homeostasis.

The moderate positive correlation between membrane conductance and membrane capacitance at the higher medium conductivity may further reflect coordinated remodeling of the plasma membrane during infection. Specific membrane capacitance is sensitive to membrane morphology, including microvilli density, surface folding, and lipid composition [46,47]. *A. phagocytophilum* is known to extensively co-opt host membrane trafficking and lipid metabolism, including cholesterol acquisition and recruitment of membrane components to the pathogen-occupied vacuole [48,49]. Such infection-induced restructuring of the plasma membrane could simultaneously alter both its capacitive (morphological) and conductive (permeability) characteristics, producing the positive coupling observed. The fact that these correlations strengthen at higher medium conductivity may reflect improved sensitivity of the dielectric spectrum to membrane-localized polarization phenomena under conditions where the contrast between cytoplasm and suspending medium is reduced [43,50].

**Figure 5:**
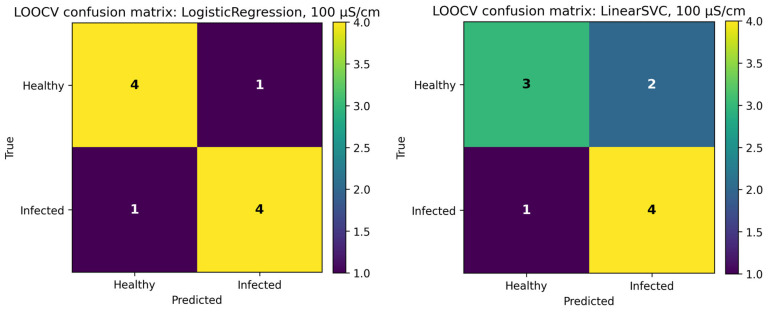
Leave-one-out cross-validation confusion matrices for infection-status classification at 100 µS/cm medium conductivity. Logistic regression results are shown on the left, and linear support vector machine results are shown on the right. Rows indicate the true class, and columns indicate the predicted class, where 0 represents uninfected HL-60 samples, and 1 represents *A. phagocytophilum*-infected HL-60 samples.

**Figure 6:**
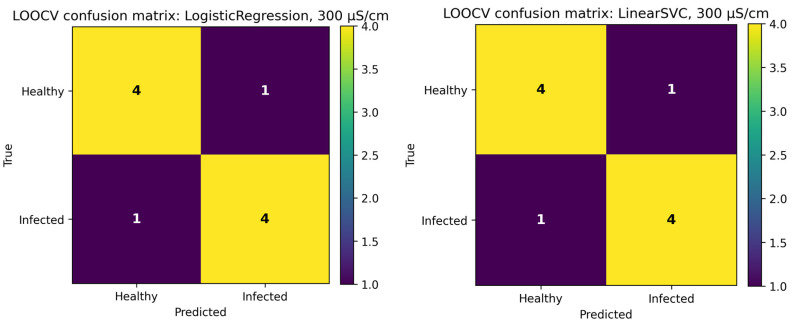
Leave-one-out cross-validation confusion matrices for infection-status classification at 300 µS/cm medium conductivity. Logistic regression results are shown on the left, and linear support vector machine results are shown on the right. Rows indicate the true class, and columns indicate the predicted class, where 0 represents uninfected HL-60 samples, and 1 represents *A. phagocytophilum*-infected HL-60 samples.

Next, machine learning models were evaluated to assess whether these dielectric features could distinguish infected from uninfected cells under each conductivity condition. Figure 5 and Figure 6 summarize LOOCV confusion matrices for logistic regression and linear support vector machine models at 100 µS/cm and 300 µS/cm, respectively. At 100 µS/cm, both classifiers show moderate performance. The linear SVM correctly identifies 3 of 5 healthy cells and 4 of 5 infected cells, while logistic regression correctly identifies 4 healthy and 4 infected cells. Misclassifications occur in both directions, reflecting the overlap observed in the feature distributions.

At 300 µS/cm, both models show slightly improved and more balanced classification behavior. The linear SVM correctly classifies 4 of 5 healthy cells and 4 of 5 infected cells, while logistic regression produces the same pattern of correct predictions. These results are consistent with the visual patterns observed in Figure 1, Figure 2, Figure 3 and Figure 4 and suggest that higher medium conductivity enhances the separability of infected and uninfected cells in the dielectric feature space.

This study has several limitations. The dataset comprises 20 dielectric measurements obtained from technical replicates of cell populations, which limits statistical power and restricts generalizability; accordingly, all findings should be interpreted as exploratory. No independent external validation set was available; therefore, leave-one-out cross-validation was used as an internal exploratory evaluation and cannot be interpreted as evidence of predictive performance. Furthermore, measurements were derived from a single biological replicate per condition, with five technical replicates used to assess measurement reproducibility rather than biological variability. As such, the reported differences between *A. phagocytophilum*-infected and uninfected cells reflect technical-level reproducibility rather than population-level biological variation. Measurements were obtained from a single immortalized cell line under controlled in vitro conditions and at two medium conductivity levels, and the study did not examine multiple infection time points or multiplicities of infection (MOI), which may limit generalizability across cell types, donor backgrounds, infection stages, and infection burdens. Freshly cultured, non-cryopreserved cells will need to be used to confirm that the observed dielectric patterns are robust across cell-preparation conditions. In addition, the present study did not include disease-specificity controls, such as HL-60 cells infected with other granulocytic pathogens. Therefore, the observed dielectric differences should be interpreted as infection-associated measurement-level changes under the tested conditions, rather than as markers specific to *A. phagocytophilum*. Nevertheless, the results are encouraging and support future studies that incorporate multiple biological replicates, additional cell lines, and a wider range of experimental conditions to validate and generalize the findings.

The results of this study indicate that dielectric features can distinguish infected from uninfected samples under the tested conditions. The separation is more apparent at 300 µS/cm, where both cytoplasmic conductivity and specific membrane conductance contribute strongly to discrimination. These findings are consistent with prior work showing that dielectric measurements are sensitive to changes in cellular state and membrane properties [16,51,52].

## 4. Conclusions

This pilot study provides preliminary measurement-level evidence that dielectric properties of HL-60 cells, particularly membrane-related features such as specific membrane conductance, may differ between *A. phagocytophilum*-infected and uninfected samples under controlled in vitro conditions. Because the dataset was small and based on technical replicates, these findings should be interpreted cautiously and not as biological or diagnostic validation. Future studies will include independent biological replicates, external validation, additional cell types or primary granulocytes, and comparative pathogen controls to assess the robustness, generalizability, and specificity of these dielectric patterns.

## Figures and Tables

**Table 1:** Exploratory statistical comparison of dielectric features between uninfected and *A. phagocytophilum*-infected HL-60 samples at each medium conductivity. Two-sided Mann–Whitney U tests were used for group comparisons. Adjusted *p*-values were calculated using the Benjamini–Hochberg procedure within each conductivity condition. Effect sizes are reported as Cohen’s d and rank-biserial correlation with bootstrap 95% confidence intervals. Because the measurements are technical replicates from a single biological preparation per condition, the results should be interpreted as measurement-level separation rather than evidence of biological generalizability.

Conductivity (µS/cm)	Feature	Direction in Infected vs. Uninfected	U	BH-Adjusted *p*-Value	Cohen’s d [95% CI]	Rank-Biserial r [95% CI]
100	Cytoplasmic conductivity	Lower	13	*p* = 1.00000 BH *p* = 1.00000	−0.30 [−1.98, 1.13]	−0.04 [−0.84, 0.68]
100	Specific membrane conductance	Higher	2	*p* = 0.03175 BH *p* = 0.09524	1.91 [0.72, 6.46]	0.84 [0.36, 1.00]
100	Specific membrane capacitance	Lower	13	*p* = 1.00000 BH *p* = 1.00000	−0.20 [−1.82, 1.11]	−0.04 [−0.76, 0.76]
300	Cytoplasmic conductivity	Lower	24	*p* = 0.01587 BH *p* = 0.02381	−1.51 [−3.83, −1.03]	−0.92 [−1.00, −0.52]
300	Specific membrane conductance	Higher	1	*p* = 0.01587 BH *p* = 0.02381	2.49 [1.46, 5.67]	0.92 [0.52, 1.00]
300	Specific membrane capacitance	Higher	3	*p* = 0.05556 BH *p* = 0.05556	1.20 [0.12, 3.61]	0.76 [0.20, 1.00]

Note. BH = Benjamini–Hochberg; CI = confidence interval. The results are based on technical replicates from a single biological preparation per condition and should therefore be interpreted as measurement-level separation rather than biological generalizability.

## Data Availability

The datasets presented in this article are not readily available because the data are part of an ongoing study. Requests to access the datasets should be directed to the corresponding author.
